# Determination of Mineral Composition and Phenolic Content and Investigation of Antioxidant, Antidiabetic, and Antibacterial Activities of *Crocus sativus* L. Aqueous Stigmas Extracts

**DOI:** 10.1155/2021/7533938

**Published:** 2021-05-29

**Authors:** Lemiae Zaazaa, Hanae Naceiri Mrabti, Abdelaziz Ed-Dra, Khaoula Bendahbia, Hind Hami, Abdelmajid Soulaymani, Mohammed Ibriz

**Affiliations:** ^1^Laboratory of Plant and Animal Production, Agro-industries, Faculty of Sciences, IBN Tofail University, B.P. 133 14000, Kenitra, Morocco; ^2^Laboratory of Pharmacology and Toxicology, Bio Pharmaceutical and Toxicological Analysis Research Team, Faculty of Medicine and Pharmacy, Mohammed V University in Rabat, BP 6203, Rabat, Morocco; ^3^Laboratory of Chemistry-Biology Applied to the Environment, Team of Microbiology and Health, Moulay Ismail University, Faculty of Sciences, BP. 11201, Zitoune, Meknes, Morocco; ^4^Laboratory of Biology and Health, Faculty of Sciences, IBN Tofail University, B.P. 133 14000, Kenitra, Morocco

## Abstract

The aim of this study is to investigate the *in vitro* antioxidant, antidiabetic, and antibacterial activities of Moroccan and Italian *Crocus sativus* (L.) stigmas extracts. The antioxidant activity was evaluated by DPPH radical scavenging assay, and the results showed that the Moroccan extract has a powerful antioxidant activity with an IC_50_ of 0.32 ± 0.059 *μ*g/mL compared to the Italian extract (IC_50_ of 3.14 ± 0.021 *μ*g/mL). Additionally, the antidiabetic activity was evaluated by using alpha-amylase and alpha-glucosidase inhibition assay, and both extracts showed significant antidiabetic activity. However, the antibacterial activity was evaluated by the disc diffusion method to determine the inhibitory diameters and microplate dilutions method to determine the minimum inhibitory concentration. Our findings revealed that both Moroccan and Italian extracts were more effective against Gram-positive than Gram-negative bacteria. From this study, we can conclude that the studied extracts of *C. sativus* are rich in natural compounds and could have a broad application in the pharmaceutical, food, and medical fields.

## 1. Introduction

Natural products have been used since antiquity as remedies for the treatment of several diseases, as well as in traditional food preparations. Nowadays, the use of natural products, especially medicinal plants and spices, takes a major place in the research of new alternative or precursors to chemical drugs that are used in the treatment of several chronic diseases. Indeed, many studies have approved the antioxidant, anti-inflammatory, and anticarcinogenic, antidiabetic, and antimicrobial activity of plants extracts and spices [1–5].

*Crocus sativus* L. is an herbaceous species belonging to the Iridaceae family, commonly known as saffron. Harvest, processing, handling, drying, packing, and storage are the stages involved in its production [6]. The flowering of *C. sativus* occurs, in an extremely short period, only once a year, and its harvest must be done manually [7], and for the production of 1 kg of saffron (dry stigmas), nearly 158,000 flowers are needed [8], which makes red dried stigmas of *C. sativus* one of the most expensive culinary herbs in the world [9].

Saffron has been cultivated in Morocco for centuries in the Taliouine region precisely over an area of 565 ha, and more recently in the region of Taznakht (province of Ouarzazate) on an area of 105 ha [10, 11]. Its cultivation is considered an important source of employment and income for the local population. However, its aroma, flavor, and good quality have given it national and international appreciation [12, 13].

Since ancient times, saffron has been widely used as a spice for culinary purposes and food coloring, especially in the food industries, and it has been used in traditional medicine, textiles, and cosmetics. Due to its healing properties, saffron has been used to treat various disorders such as insomnia, liver disease, digestive disorders, and menstruation disorders, and it was also used as an aphrodisiac, antidepressant, and stimulant [13, 14]. However, the biological activities of Moroccan *C. sativus* are still not well studied. Thus, in order to fill this gap, we evaluated the chemical and biological activities of Moroccan and Italian *C. sativus* extracts.

## 2. Materials and Methods

### 2.1. Plant Material

The plant of *C. sativus* was obtained from Taliouine region in Morocco during flowering stage and from Italy. After harvest, the plant material (stigma) was dried at room temperature (22°C) in the open air and protected from light and heat. Then, they were crushed for extraction.

### 2.2. Preparation of the Saffron Extracts

Five grams of the dried plant material was infused in 100 ml of boiling distilled water for 15 min, then filtered, and evaporated under vacuum at 40°C using a rotary evaporator. The extracts were cooled and stored in the refrigerator at 4°C until use.

### 2.3. Determination of Total Phenolic Compounds

The amount of total phenolic contents was determined according to Folin-Ciocalteu method [15], with some modifications. Briefly, a volume of 100 *μ*l of each extract was introduced using a micropipette into test tubes, followed by the addition of 1000 *μ*l of the Folin-Ciocalteu reagent (0.2 N). After 10 min of incubation at room temperature, 800 *μ*l of sodium carbonates (Na_2_CO_3_) (75 g/l) has been added, and the solutions were kept in the dark for 30 minutes at room temperature. The absorbance of each solution was measured by spectrophotometer at 765 nm. The total phenolic content was expressed as milligrams gallic acid equivalents per Gram of dry weight of extract (mg GAE/g extract).

### 2.4. Determination of Flavonoid Contents

The total flavonoids were determined using a colorimetric method [16] with few modifications. Briefly, 250 *μ*l of the extract was mixed with 1000 *μ*l of distilled water, and then 75 *μ*l of a 15% sodium nitrite (NaNO_2_) solution was added. After 6 minutes of incubation at room temperature, 75 *μ*l of 10% aluminum chloride (AlCl_3_) was added. After 6 minutes of incubation at room temperature, 1000 *μ*l of sodium hydroxide (NaOH) (4%) was added. The total volume is made up to 2500 *μ*l of distilled water. The solution was mixed and left to stand for 15 min. The absorbance was measured at 510 nm against a blank. Total flavonoid contents (TFCs) were expressed as mg Catechin equivalent per Gram of dry weight of extract (mg CE/g extract).

### 2.5. Determination of Mineral Content by ICP-AES

Inductively coupled plasma atomic emission spectroscopy (ICP-AES) was used for rapid and precise determinations of minor and major mineral content in digested powder of studied plants. Briefly, 1 g of *C. sativus* stigma was digested by heating in an oven at 500°C, and the rest of the powder was totally solubilized in 5 ml of concentrated nitric acid (HNO_3_) + 15 ml of hydrochloric acid (HCl). After complete digestion, the mixed samples were cooled at room temperature and made up to a final volume 100 ml with ultrapure water. Digestion was then analyzed in duplicate, and concentrations of trace metal elements were measured directly using Inductively Coupled Plasma-Optical Emission Spectrometry (ICP-OES, Thermo Scientific). The optimal instrumental conditions are maintained at 12 l/min for the stable plasma gas flow rate, and the auxiliary and the nebulizer gas flow rate were kept at 1 and 0.7 l/min, respectively. Indeed, the sample flow rate was 1.5 ml/min, and the Radio Frequency (RF) power was 1200 W.

### 2.6. Determination of Total Antioxidant Capacity

The antioxidant activity was measured using the DPPH method. The purplish hue of the free and stable radical DPPH (2,2-diphenyl-picryl-hydrazyl) is absorbed at 517 nm. This radical is reduced and turns yellow in the presence of antioxidant molecules. Briefly, 0.5 ml of each sample concentration was mixed with 0.5 ml of methanolic solution of DPPH (0.04 g/l) and then incubated in the dark at room temperature for 30 min [17]. Absorbance was measured at *λ* = 517 nm using a spectrophotometer. The calculation of the percentage inhibition of DPPH is carried out by applying the following equation:

(1) $$\begin{array}{c} \%\text{inhibition}=\frac{\left(\text{A blank}-\text{A sample}\right)}{\text{A blank}}\times 100, \end{array}$$

A blank: absorbance value of the DPPH blank sample A sample: absorbance value of the extract solution.

Ascorbic acid was used as a positive control, and the percent inhibition versus extract concentration curve was used to measure concentrations of 50% inhibition (IC_50_).

### 2.7. *In Vitro* Antidiabetic Activity Assessment

#### 2.7.1. *α*-Amylase Inhibition Assay

The *α*-amylase inhibition assay was conducted by reacting different concentrations of *C. sativus* stigma aqueous extracts with *α*-amylase enzyme and starch solution, as previously described [18], with slight modifications. Briefly, 250 *µ*l of extract was mixed with 250 *µ*l of *α*-amylase (240 U/ml, in 0.02 M phosphate buffer solution (PBS), pH 6.9, with 0.006 M NaCl) and incubated at 37°C for 10 min. After incubation, 250 *µ*l of 1% (w/v) soluble starch (in 0.02 M PBS, pH 6.9) was added, and the obtained mixture was subjected to a second incubation at 37°C for 30 min. Then, 250 *µ*l of DNS color reagent was added, and the reaction was stopped by heating in a boiling water bath for 10 min. After cooling to room temperature, the mixture has been diluted with 2 ml of PBS, and the absorbance was measured at 540 nm.

#### 2.7.2. *α*-Glucosidase Inhibition Assay

Different concentrations of extracts were prepared in distilled water. However, the *α*-glucosidase enzyme (0.1 U/ml) and substrate *p*-Nitrophenyl-*α*-D-glucopyranoside (*p*-NPG, 1 mM) were dissolved in PBS (0.1 M, pH 6.7). Afterward, 150 *μ*l of each extract concentration was preincubated with 100 *μ*l of enzyme at 37°C for 10 min, and then 200 *μ*l of substrate was added to the reaction mixture. The enzymatic reaction was conducted at 37°C for 30 min and stopped by adding 1 ml of Na_2_CO_3_ (1 M). All samples were analyzed in triplicate with different concentrations to determine the IC_50_ values, and the absorbance was measured at 405 nm. The inhibition percentage (%) was calculated by the following equation for both assays:

(2) $$\begin{array}{c} \text{inhibition}\left(\text{\%}\right)=\frac{\left(\text{AC}-\text{ACb}\right)-\left(\text{AS}-\text{ASb}\right)}{\text{AC}-\text{ACb}}\times 100. \end{array}$$

Absorbance was abbreviated as follows: AC (control), AC_b_ (control blank), AS (sample), and AS (sample blank).

### 2.8. Determination of Antibacterial Activity

The antibacterial activity of *C. sativus* extracts was evaluated against four Gram-negative bacteria, including *E. coli* ATCC 25922, *Salmonella typhimurium* ATCC 14028, *Pseudomonas aeruginosa* ATCC 15442, and *Klebsiella pneumonia* ATCC 43816, and two Gram-positive bacteria, including *Staphylococcus aureus* ATCC 25923 and *Listeria monocytogenes* ATCC 13932. Before conducting experiments, the bacterial cultures were inoculated on Tryptone Soya Agar (TSA) (Biokar, Beauvais, France) from the frozen stock and incubated at 37°C during 24 h.

#### 2.8.1. Disc Diffusion Assay

The antibacterial activity of *C. sativus* extracts was evaluated by disc diffusion method according to the protocol described previously [19], with a few modifications. Briefly, a bacterial suspension equivalent to 0.5 McFarland (10^8^ cfu/mL) was prepared and swabbed on the surface of Mueller-Hinton agar (MHA) (Biokar, Beauvais, France) plates. Afterwards, a 6 mm diameter filter disc (Whatman No. 4) soaked in 20 *µ*l of each extract (1 g/ml) was deposited on the surface of MHA plate. Sterile distilled water (10 *µ*l) and gentamicin (30 *µ*g) were used, respectively, as negative and positive controls of this study. The plates were left for 15 minutes until the absorption of extracts, and then, they were incubated at 37°C for 18–24 h. After incubation, the inhibition diameter of each extract was measured in millimeters (disk included).

#### 2.8.2. Determination of MIC and MBC

Determination of Minimum Inhibitory Concentration (MIC) and Minimum Bactericide Concentration (MBC) was conducted according to the microplate method following the protocol described previously [20]. Briefly, decreasing concentration of extracts was prepared, inoculated by bacterial inoculum, and incubated at 37°C for 24 h. After incubation, the wells correspond to the lower concentration and do not present visible growth of bacteria were considered as the MIC. The MBC was determined by subculturing samples from the wells on TSA (Biokar, Beauvais, France) and incubated at 37°C for 24 h. The lower concentration that did not present any growth on TSA was considered as the MBC.

### 2.9. Statistical Analysis

Microsoft Excel (New York, USA) was used for data analysis. Experiments were performed in triplicate, and the results were presented as means ± standard error. Student's test (*P* < 0.05) was used to evaluate the statistical difference between the studied extracts.

## 3. Results and Discussion

### 3.1. Determination of Total Phenolic and Flavonoid Contents

The total phenolic contents (TPC) and the total flavonoid contents (TFC) of the aqueous extracts of *C. sativus* are presented in Table 1. The results showed that the Moroccan extract contained a high TPC (72.47 ± 1.62 mg GAE/g extract) compared to the Italian extract (31.47 ± 0.18 mg GAE/g extract). Similarly, flavonoids content showed higher amounts in Moroccan extract (51.47 ± 0.32 mg CE/g extract) than Italian extract (22.61 ± 1.25 mg CE/g extract). Statistical analysis showed that the difference in TPC and TFC between the Moroccan and Italian extracts is statistically significant (*P* < 0.05). These can be explained by the difference in ecological conditions, such as soil type, microclimatic conditions, geographic position, sampling site, age, and vegetative stage of plants and fruits [21–23]. Phenolic compounds are natural antioxidants occurring in green vegetables, fruits, and oils [24, 25]. Some phenolic compounds have been reported to possess anti-inflammatory, antiviral, and anticancer activities [24, 26]. These compounds have gained attention because of the protective effect played by antioxidants in many pathological diseases, in which oxidative stress is implicated [4].

### 3.2. Determination of Mineral Content

Mineral elements are involved in important biological functions of the cells. The mineral content, including the macroelements (Ca, P, K, Mg, and Na), the microelements (S, Co, Fe, B, Cu, Zn, Mn, and V), and the heavy metals (Cd, Pb, Ni, Mo, Cr, and AS), was determined in both Moroccan and Italian *C. sativus* extracts, and the results are presented in Table 2. Our results indicate that potassium (K) was the most abundant macroelement in both extracts, with concentration ranging from 55.32 g/kg in the Moroccan extract to 53.021 g/kg in the Italian extract. Sulfur was the most abundant microelement with a concentration of 5.4 g/kG for the Moroccan extract and 4.8 g/Kg for the Italian extract. For the other macro- and microelements, interesting differences were recorded between the Moroccan and Italian extracts; these differences can be explained by climatic conditions, pedologic condition, crop cycle length, drying method, irrigation, corm planting, and dimension [27].

### 3.3. Antioxidant Activity

In a preliminary investigation of the antioxidant potential of *C. sativus* extracts, we investigated the radical scavenging activity by DPPH. The property of the DPPH• radical, which is remarkably stable, is due mainly to the steric hindrance around the divalent nitrogen and, to a lesser extent, to the “push-pull” effect of electron donor and acceptor (diphenylamino and picryl group, respectively) onto the divalent N [28]. During the reaction, free radicals accept a single pair of electrons or hydrogen atoms and converge from purple to yellow color. The scavenging effect of *C. sativus* extracts and the ascorbic acid on DPPH radical was expressed by IC_50_, which is presented in Table 3. Our results showed that the Moroccan extract presents the higher antioxidant power (IC_50_ = 0.32 ± 0.059 *μ*g/ml) compared with the Italian extract (IC_50_ = 3.14 ± 0.021 *μ*g/ml). Interestingly, the antioxidant activity of Moroccan extract showed a comparable IC_50_ with Ascorbic acid (0.29 ± 0.003 *µ*g/ml). In fact, previous studies have investigated the DPPH radical scavenging effect of flowers, petals, and leaves of *C. sativus* [12, 24, 29], while few papers have described the antioxidant activity of stigmas [30]. Additionally, the difference between the antioxidant activity of Moroccan and Italian extracts could be due to the difference in their phenolic and flavonoid contents. Indeed, many studies previously mentioned that phenolic and flavonoid compounds have remarkably antioxidant properties in both *in vitro* and *in vivo* systems [28, 31, 32].

### 3.4. Antidiabetic Activity

The inhibitory activity of both Moroccan and Italian extracts against enzymes *α*-glucosidase and *α*- amylase was evaluated, and the obtained results are summarized in Table 4. The results showed that the Moroccan extracts exhibited the higher inhibitory activity against *α*-glucosidase and *α*-amylase, with an IC_50_ of 75.25 ± 1.05 *μ*g/ml and 687.12 ± 0.21 *μ*g/ml, respectively, while the Italian extract exhibited the lower inhibitory activity with and IC_50_ of 97.83 ± 1.03 *μ*g/ml against *α*-glucosidase and an IC_50_ of 720.05 ± 1.01 *μ*g/ml against *α*-amylase. The potency of *C. sativus* aqueous extracts to inhibit the enzymes *α*-glucosidase and *α*-amylase suggests that the compounds possessing antidiabetic activity are extracted in water, supporting the traditional use of aqueous extract of *C. sativus* in the treatment of diabetes. In fact, many previous studies have demonstrated the hypoglycemic effect of phenolic compounds, by improving postprandial blood glucose levels, acute insulin secretion, and insulin sensitivity, since limiting the rate of glucose absorption from the intestines into the bloodstream was considered the only strategy that could help prevent diabetes [33].

### 3.5. Antibacterial Activity

**Figure 1:**
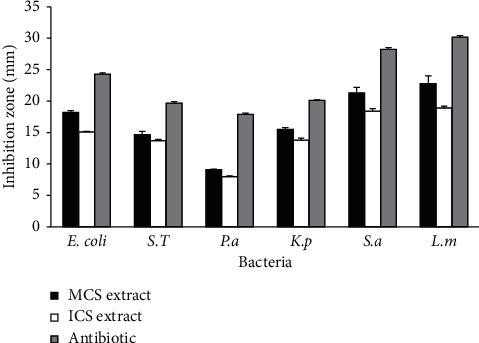
Inhibition zones of aqueous extracts of C. sativus against the 4 tested bacteria. MCS extract: Moroccan *C. sativus* extract; ICS extract: Italian *C. sativus* extract; *E. coli*: *Escherichia coli* ATCC 25922; S.T: *Salmonella typhimurium* ATCC 14028; P.a: *Pseudomonas aeruginosa* ATCC 15442; K.p: *Klebsiella pneumoniae* ATCC 43816; S.a: *Staphylococcus aureus* ATCC 25923; L.m: *Listeria monocytogenes* ATCC 13932.

The antibacterial activity of aqueous extracts of *C. sativus* was performed against six bacterial strains belonging to Gram-negative and Gram-positive bacteria, by disc diffusion assay and broth dilution method. The results of disc diffusion assay showed that Moroccan *C. sativus* extract creates an inhibition diameter ranging from 21.3 ± 0.9 mm to 22.8 ± 1.2 mm against Gram-positive bacteria and from 9.1 ± 0.1 mm to 18.2 ± 0.3 mm against Gram-negative bacteria (Figure 1), while Italian extract creates an inhibition zone ranged from 18.4 ± 0.4 mm to 22.8 ± 1.2 mm against Gram-positive bacteria and from 8.0 ± 0.1 mm to 15.1 ± 0.1 mm against Gram-negative bacteria (Figure 1). However, results of broth dilution method showed that Moroccan *C. sativus* extract had a MIC value of 3.9 mg/ml for Gram-positive bacteria, and MIC ranged from 15.62 mg/ml to 62.5 mg/ml for Gram-negative bacteria (Table 5). Likewise, Italian *C. sativus* extract had a MIC value of 7.81 mg/ml for Gram-positive bacteria, and the value was between 31.25 mg/ml and 125 mg/ml for Gram-negative bacteria (Table 5). In addition, our results demonstrated that both *C. sativus* extracts had a bactericidal effect with a ratio of MBC/MIC<4.

So far, some worldwide studies have reported the antimicrobial activity of *C. sativus* extracts. In a study performed previously in Morocco, Jadouali and colleagues showed that leave extracts of *C. sativus* had a great antibacterial activity against *Listeria* sp. [29]. Also, Vahidi and his group studied the antimicrobial activity of *C. sativus* extracts harvested from Khorasan in Iran and prepared them with different solvents against various strains of bacteria and fungi [34]. However, Asgarpanah et al. reported the antimicrobial effect of methanol extract prepared from different parts of *C. sativus* harvested from southern Khorasan province in Iran against various strains of bacteria and fungi [35]. In Turkey, ethanol and aqueous extracts of *C. sativus* showed interesting antibacterial effect against mastitis pathogens [36]. And, Muzaffar et al. showed the high antibacterial activity of methanolic and petroleum ether extracts of *C. sativus* collected from India [37].

Our results showed that aqueous extract of *C. sativus* is more effective against Gram-positive bacteria than Gram-negative. This difference could be due to the difference in the cell wall structure of the tested bacteria. Indeed, the susceptibility of Gram-positive bacteria to *C. sativus* extract in comparing with Gram-negative bacteria might be explained by the higher susceptibility of its cell wall to the chemical components containing extracts. Nevertheless, the lower sensitivity of Gram-negative bacteria can be explained by the difficulty of the extract components to diffuse through the hydrophilic barrier of the cell wall [34]. Additionally, our results showed that the antibacterial activity of *C. sativus* extract originating from Morocco is higher than that originating from Italy. This difference could be linked to the difference in the chemical composition of the studied extracts. In fact, the richness of Moroccan extract with flavonoids and phenolic compounds may strengthen its antibacterial activity [35, 38].

## 4. Conclusion

Morocco is a country that has rich plant resources with a high diversity of medicinal plants that are used in the treatment and prevention of several illnesses. From this study, we conclude that Moroccan *C. sativus* stigmas extract has a great antioxidant, antimicrobial, and antidiabetic properties and is rich in mineral elements. Therefore, it can be used as a source of bioactive compounds and in the treatment of infectious diseases caused by resistant bacteria, in addition to the broad applications in food and medical fields. However, based on the results of this study, further *in vivo* and *ex vivo* confirmatory tests for effective extracts and subfractions of *C. sativus* are recommended.

## Figures and Tables

**Table 1:** Total phenolic and flavonoid contents of *C. sativus* aqueous extracts.

	TPC (mg GAE/g extract)	TFC (mg CE/g extract)
Moroccan extract	72.47 ± 1.62^a^	51.47 ± 0.32^a^
Italian extract	31.47 ± 0.18^b^	22.61 ± 1.25^b^

Data are expressed as mean ± SD (*n* = 3). TFC: total flavonoid content; TPC: total phenolic content; mg CE/g extract: mg catechin equivalent per gram of extract; mg GAE/g extract: mg Galic Acid equivalent per gram of extract. Different letters in the same row represent significant differences at *P* < 0.05.

**Table 2:** Mineral contents in mg/Kg dw of *C. sativus* aqueous extracts.

Moroccan extract		Italian extract
*Macroelements*		
Ca	589.12	512.23
Mg	2849.17	2540.87
P	1030	914.45
Na	940	841.23
K	55320	53021

*Microelements*		
S	5402.12	4801.27
B	17.5	10.02
Cu	9.11	8.17
Mn	68.15	57.15
Zn	94.205	81.21
V	3.124	2.85
Co	0.498	0.123
Cr	2.31	1.24
Fe	185.24	178.14

*Heavy metals*		
Cd	0.014	0.1
Pb	0.798	0.254
Ni	ND	ND
Mo	ND	ND
Cr	2.31	1.11
As	0.001	ND

ND: not detected.

**Table 3:** Free radical scavenging activity of *C. sativus* extract.

Antioxidant activity	Moroccan extract	Italian extract	Ascorbic acid
IC_50_ (*μ*g/mL)	0.32 ± 0.059^a^	3.14 ± 0.021^b^	0.29 ± 0.003^a^

Data are expressed as mean ± SD (*n* = 3). Different letters in the same row represent significant differences at *P* < 0.05.

**Table 4:** Digestive enzyme inhibition activity (*α*-glucosidase and *α*-amylase) of *C. sativus* aqueous extracts.

Studied agents	IC_50_ (*μ*g/ml)	
	*α*-Amylase	*α*-Glucosidase
Moroccan extract	687.12 ± 0.21^b^	75.25 ± 1.05^a^
Italian extract	720.05 ± 1.01^c^	97.83 ± 1.03^b^
Acarbose	334.40 ± 1.24^a^	119.41 ± 0.02^c^

Data are expressed as mean ± SD (*n* = 3). Different letters in the same row represent significant differences at *P* < 0.05.

**Table 5:** Results for MIC and MBC for aqueous extract of *C. sativus*.

Bacteria	Gram	Moroccan extract (mg/mL)			Italian extract (mg/mL)		
		MIC	MBC	R	MIC	MBC	r
*E. coli* ATCC 25922	−	15.62	15.62	1	31.25	31.25	1
*S. typhimurium* ATCC 14028	−	31.25	31.25	1	62.5	62.5	1
*P. aeruginosa* ATCC 15442	−	62.5	125	2	125	125	1
*K. pneumoniae* ATCC 43816	−	31.25	31.25	1	31.25	62.5	2
*S. aureus* ATCC 25923	+	3.9	3.9	1	7.81	7.81	1
*L. monocytogenes* ATCC 13932	+	3.9	7.81	2	7.81	15.62	2

The ratio (r): MBC/MIC.

## Data Availability

All used data in this study are included within the manuscript.

## References

[1] Abdallah E. M., Elsharkawy E. R., Ed-Dra A. (2016). Biological activities of methanolic leaf extract of *Ziziphus mauritiana*. *Bioscience Biotechnology Research Communications*.

[2] Ed-Dra A., Filali F. R., Lo Presti V. (2020). Chemical composition, antioxidant capacity and antibacterial action of five Moroccan essential oils against Listeria monocytogenes and different serotypes of *Salmonella enterica*. *Microbial Pathogenesis*.

[3] Mrabti H. N., Jaradat N., Kachmar M. R. (2019). Integrative herbal treatments of diabetes in Beni Mellal region of Morocco. *Journal of Integrative Medicine*.

[4] Mrabti H. N., Sayah K., Jaradat N. (2018). Antidiabetic and protective effects of the aqueous extract of *Arbutus unedo* L. in streptozotocin-nicotinamide-induced diabetic mice. *Journal of Complementary and Integrative Medicine*.

[5] Embuscado M. E. (2015). Spices and herbs: natural sources of antioxidants—a mini review. *Journal of Functional Foods*.

[6] Cosano I., Pintado C., Acevedo O. (2009). Microbiological Quality of saffron from the main producer countries. *Journal of Food Protection*.

[7] Sano K., Himeno H. (1987). *In vitro* proliferation of saffron (*Crocus sativus* L.) stigma. *Plant Cell, Tissue and Organ Culture*.

[8] Alonso G. L., Salinas M. R., Sánchez-Fernández M. A., Garijo J. (2000). Note. Physical parameters in controlling saffron quality/Nota. Algunos parámetros físicos del control de calidad del azafrán. *Food Science and Technology International*.

[9] Serghini M. A., Lagram K., Ben El Caid M. Saffron (*crocus sativus*): current state of scientific research.

[10] Lage M., Cantrell C. L. (2009). Quantification of saffron (*Crocus sativus* L.) metabolites crocins, picrocrocin and safranal for quality determination of the spice grown under different environmental Moroccan conditions. *Scientia Horticulturae*.

[11] El Aymani I., Qostal S., Mouden N. (2019). Fungi associated with saffron (*Crocus sativus*) in Morocco. *Plant Cell Biotechnology and Molecular Biology*.

[12] Jadouali S. M., Atifi H., Mamouni R. (2019). Chemical characterization and antioxidant compounds of flower parts of Moroccan *Crocus sativus* L. *Journal of the Saudi Society of Agricultural Sciences*.

[13] Mzabri I., Addi M., Berrichi A. (2019). Traditional and modern uses of saffron (*Crocus sativus*). *Cosmetics*.

[14] Schmidt M., Betti G., Hensel A. (2007). Saffron in phytotherapy: pharmacology and clinical uses. *Wiener Medizinische Wochenschrift*.

[15] Nickavar B., Alinaghi A., Kamalinejad M. (2008). Evaluation of the antioxidant properties of five *Mentha* species. *Iranian Journal of Pharmaceutical Research*.

[16] Dewanto V., Wu X., Liu R. H. (2002). Processed sweet corn has higher antioxidant activity. *Journal of Agricultural and Food Chemistry*.

[17] Mrabti H. N., Bouyahya A., Ed-Dra A. (2021). Polyphenolic profile and biological properties of *Arbutus unedo* root extracts. *European Journal of Integrative Medicine*.

[18] Hashim A., Khan M. S., Khan M. S., Baig M. H., Ahmad S. (2013). Antioxidant and *α*-amylase inhibitory property of *phyllanthus virgatus* L.: an *in vitro* and molecular interaction study. *BioMed Research International*.

[19] Ed-Dra A., Filai F. R., Bou-Idra M. (2018). Application of *Mentha suaveolens* essential oil as an antimicrobial agent in fresh Turkey sausages. *Journal of Applied Biology & Biotechnology*.

[20] Chebaibi A., Marouf Z., Rhazi-Filali F., Fahim M., Ed-Dra A. (2016). Évaluation du pouvoir antimicrobien des huiles essentielles de sept plantes médicinales récoltées au Maroc. *Phytothérapie*.

[21] Aryal S., Baniya M. K., Danekhu K., Kunwar P., Gurung R., Koirala N. (2019). Total Phenolic content, Flavonoid content and antioxidant potential of wild vegetables from western Nepal. *Plants*.

[22] Hue S. M., Boyce A. N., Somasundram C. (2012). Antioxidant activity, phenolic and flavonoid contents in the leaves of different varieties of sweet potato (“ipomoea batatas”). *Australian Journal of Crop Science*.

[23] Siatka T., Kašparová M. (2010). Seasonal variation in total phenolic and flavonoid contents and DPPH scavenging activity of *Bellis perennis* L. flowers. *Molecules*.

[24] Huang W. Y., Cai Y. Z., Zhang Y. (2010). Natural phenolic compounds from medicinal herbs and dietary plants: potential use for cancer prevention. *Nutrition and Cancer*.

[25] Lin J.-Y., Tang C.-Y. (2007). Determination of total phenolic and flavonoid contents in selected fruits and vegetables, as well as their stimulatory effects on mouse splenocyte proliferation. *Food Chemistry*.

[26] Zhang L., Ravipati A. S., Koyyalamudi S. R. (2011). Antioxidant and anti-inflammatory activities of selected medicinal plants containing phenolic and flavonoid compounds. *Journal of Agricultural and Food Chemistry*.

[27] Cardone L., Castronuovo D., Perniola M., Cicco N., Candido V. (2020). Saffron (*Crocus sativus* L.), the king of spices: an overview. *Scientia Horticulturae*.

[28] Mrabti H. N., Marmouzi I., Sayah K. (2017). *Arbutus unedo* L. aqueous extract is associated with *in vitro* and *in vivo* antioxidant activity. *Journal of Materials and Environmental Science*.

[29] Jadouali S. M., Atifi H., Bouzoubaa Z. (2018). Chemical characterization, antioxidant and antibacterial activity of Moroccan Crocus sativus L petals and leaves. *Journal of Materials and Environmental Sciences*.

[30] Karimi E., Oskoueian E., Hendra R., Jaafar H. Z. E. (2010). Evaluation of *Crocus sativus* L. stigma phenolic and flavonoid compounds and its antioxidant activity. *Molecules*.

[31] Elsharkawy E. R., Ed-Dra A., Abdallah M., Ali A. M. H. (2018). Antioxidant, antimicrobial and antifeedant activity of phenolic compounds accumulated in *Hyoscyamus muticus* L. *African Journal of Biotechnology*.

[32] Saad F., Mrabti H. N., Sayah K. (2019). Phenolic content, acute toxicity of *Ajuga iva* extracts and assessment of their antioxidant and carbohydrate digestive enzyme inhibitory effects. *South African Journal of Botany*.

[33] Dada F. A., Oyeleye S. I., Ogunsuyi O. B. (2017). Phenolic constituents and modulatory effects of Raffia palm leaf (Raphia hookeri) extract on carbohydrate hydrolyzing enzymes linked to type-2 diabetes. *Journal of Traditional and Complementary Medicine*.

[34] Vahidi H., Kamalinejad M., Sedaghati N. (2010). Antimicrobial properties of *croccus sativus* L. *Iranian Journal of Pharmaceutical Research*.

[35] Asgarpanah J., Darabi-Mahboub E., Mahboubi A., Mehrab R., Hakemivala M. (2013). In-vitro evaluation of *Crocus sativus* L. Petals and stamens as natural antibacterial agents against food-borne bacterial strains. *Iranian Journal of Pharmaceutical Sciences*.

[36] Okmen G., Vurkun M., Arslan A., Ceylan O. (2017). The antibacterial activities of *Piper nigrum* L. against mastitis pathogens and its antioxidant activities. *Indian Journal of Pharmaceutical Education and Research*.

[37] Muzaffar S., Rather S. A., Khan K. Z. (2016). *In vitro* bactericidal and fungicidal activities of various extracts of saffron (*Crocus sativus* L.) stigmas from Jammu & Kashmir, India. *Cogent Food & Agriculture*.

[38] Acar G., Mercan Dogan N., Emin Duru M., Kıvrak I. (2010). Phenolic profiles, antimicrobial and antioxidant activity of the various extracts of *Crocus* species in Anatolia. *African Journal of Microbiology Research*.

